# Validation of the Dual-path Platform chromatographic immunoassay (DPP^®^ CVL rapid test) for the serodiagnosis of canine visceral leishmaniasis

**DOI:** 10.1590/0074-02760180206

**Published:** 2018-10-29

**Authors:** Fabiano Borges Figueiredo, Tassia Cristina Bello de Vasconcelos, Maria de Fátima Madeira, Rodrigo Caldas Menezes, Ana Nilce Silveira Maia-Elkhoury, Andreza Pain Marcelino, Guilherme L Werneck

**Affiliations:** 1 Fundação Oswaldo Cruz-Fiocruz, Instituto Carlos Chagas, Laboratório de Biologia Celular, Curitiba, PR, Brasil; 2 Prefeitura Municipal de Resende, Vigilância em Saúde, Centro de Controle de Zoonoses, Resende, RJ, Brasil; 3 Fundação Oswaldo Cruz-Fiocruz, Instituto Nacional de Infectologia, Laboratório de Vigilância em Leishmanioses, Rio de Janeiro, RJ, Brasil; 4 Fundação Oswaldo Cruz-Fiocruz, Instituto Nacional de infectologia, Laboratório de Pesquisa Clínica em Dermatozoonoses em Animais Domésticos, Rio de Janeiro, RJ, Brasil; 5 Organização Pan-Americana de Saúde, Doenças Negligenciadas, Tropicais e Transmitidas por Vetores, Doenças Transmissíveis e Determinantes Ambientais de Saúde, Duque de Caxias, RJ, Brasil; 6 Fundação Ezequiel Dias, Instituto Otávio Magalhães, Belo Horizonte, MG, Brasil; 7 Universidade do Estado do Rio de Janeiro, Instituto de Medicina Social, Departamento de Epidemiologia, Rio de Janeiro, RJ, Brasil

**Keywords:** Dual-path Platform CVL rapid test, visceral leishmaniasis, dog, diagnosis

## Abstract

BACKGROUND Visceral leishmaniasis is a major public health challenge in South America, and dogs are its main urban reservoir. OBJECTIVE Validation of the canine Dual-path Platform immunoassay for canine visceral leishmaniasis (DPP^®^ CVL) for a sample set composed of 1446 dogs from different Brazilian endemic areas. METHODS A well-defined reference standard by means of parasitological culture, immunohistochemistry, and histopathology was used. Animals were classified as asymptomatic, oligosymptomatic, or symptomatic. Sensitivity and specificity were assessed as a single set and in clinical groups. A reproducibility assessment of the tests was conducted using the Kappa (κ) index at three different laboratories (A, B, and C). FINDINGS Overall, 89% sensitivity and 70% specificity were obtained for the entire sample set. Analysis of the clinical groups showed a gradual decrease in the sensitivity and an increase in the specificity with the reduction of clinical signs in the dogs that were assessed, reaching a sensitivity of 75% (42.8-94.5%) among asymptomatic dogs and lower specificity of 56% (46.2-66.3%) among symptomatic dogs. Inter-laboratory agreement was substantial (κ_AB_= 0.778; κ_AC_= 0.645; κ_CB_= 0.711). MAIN CONCLUSIONS The test performance is somewhat dependent on canine symptomatology, but such influence was less evident than in previous studies. Favourable results for sensitivity and specificity can be obtained even in asymptomatic animals; however, caution is needed in these evaluations, and the results suggest that the immunochromatographic test may be further improved for better investigation in asymptomatic dogs. The results obtained confirm the usefulness of DPP^®^ CVL for application in serological surveys.

Visceral leishmaniasis (VL) is typically a zoonosis that affects humans and other species of domestic and wild animals, but anthroponotic transmission predominates on the Indian subcontinent and in parts of Africa.^1^ ^,^ ^2^ On the American continents, this disease is caused by *Leishmania infantum* (sin. *Leishmania chagasi*), and sand flies of the genus *Lutzomyia* are the vectors involved in its transmission.^2^ ^,^ ^3^ ^,^ ^4^ ^,^ ^5^

In South America, VL is expanding geographically and is a great challenge to public health.^2^ ^,^ ^5^ ^,^ ^6^ ^,^ ^7^ ^,^ ^8^ ^,^ ^9^ Human and canine cases have been reported in both rural and urban areas,^2^ ^,^ ^10^ ^,^ ^11^ and Brazil is among the top four countries in the world with the largest numbers of cases of this disease.^12^

In Brazil, where the transmission cycle of VL is predominantly zoonotic, dogs are the main urban reservoir.^5^ Diagnosis in this host is complex and can be conducted by means of serological, molecular and parasitological methods.^13^ Parasitological techniques are considered the reference standard,^14^ but, in endemic areas, serological tests are used as a tool in epidemiological surveys to facilitate diagnosis and decision-making.^2^ ^,^ ^15^

In 2011, the Brazilian Ministry of Agriculture Livestock and Food Supply (MAPA) registered a rapid, dual-path, chromatographic immunoassay (Dual-path Platform - DPP^®^) aimed at the diagnosis of canine visceral leishmaniasis (CVL).^15^ This test consists of a device impregnated with recombinant antigen rK28 (a chimaera combining antigens K9, K26 and K39) of *L. infantum*.^16^ Despite such characteristics and the ease of application, discussion on the accuracy of DPP persists, especially regarding its sensitivity for detection of infected asymptomatic animals.^17^

In this context, there is a need to conduct a study that addresses a representative sample of dogs from an endemic area, that uses a well-defined reference standard and blind analysis, and that follows the recommended methodological principles for the preparation and report of diagnostic accuracy studies.^18^ With this perspective, this study aimed to validate and assess the inter-laboratory concordance of the DPP^®^ CVL chromatographic immunoassay by applying it to samples of animals from different Brazilian endemic areas.

## MATERIALS AND METHODS

The present study conducted the validation of a multicentric, blind, diagnostic test of a sample set composed of 1446 dogs that were systematically selected in four municipalities located in different regions of Brazil in which VL is endemic. Sample size calculations were based on an estimated 10% CVL prevalence, 90% test sensitivity, 80% test specificity, and 5% error.

**Fig. 1:**
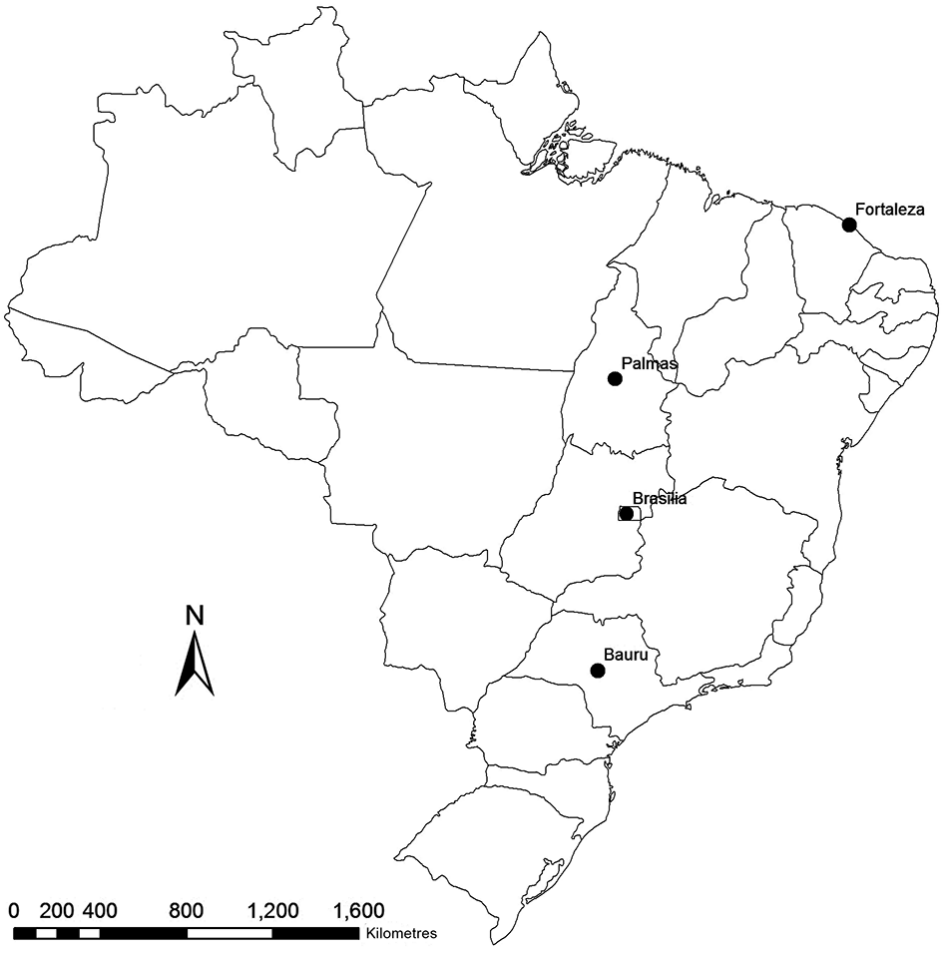
localisation of participating municipalities in each Brazilian region for the DPP® CVL validation study: Bauru, São Paulo state, Southeast region; Brasília, Federal District, Mid-West region; Palmas, Tocantins state, North region; and Fortaleza, Ceará state, Northeast region.

Participating municipalities and their respective states and regions were as follows: Bauru, São Paulo state, Southeast region; Brasília, Federal District, Mid-West region; Palmas, Tocantins state, North region; Fortaleza, Ceará state, Northeast region (Fig. 1). In each municipality, three distinct, non-continuous neighbourhoods with the highest prevalence of CVL were selected. The dogs were selected through an active, door-to-door search. The animal selection process for the study followed a systematic sampling procedure beginning with a randomly selected house, in which at least one dog was present. The subsequent house was passed, and the next one was visited until another dog was found; this process continued until there were no more houses available in each neighbourhood or until the sample size calculated for the municipality was complete.

All animals from the chosen households were evaluated and selected according to the following inclusion criteria: dogs whose owner had resided in the study region for at least six months; dogs whose owner was of legal age and qualified to sign an informed consent form; dogs aged ≥ 8 months; dogs amenable to sedation; and dogs without previous clinical assessment or laboratorial diagnosis for CVL. Exclusion criteria were as follows: pregnant bitches; aggressive dogs that could not be managed by the field team; dogs without an owner; or dogs undergoing vaccination or any anti-*Leishmania* chemotherapeutic treatment.

Clinical evaluation was performed by veterinarians on the research team, and animals were classified according to the presence of clinical signs suggestive of CVL. To this end, despite the LeishVet guidelines for classification of CVL, which consider both clinical signs and clinicopathological abnormalities,^13^ dogs enrolled in this study were evaluated exclusively by the clinical criteria due to the operational impossibility of performing pathological analyses for all animals in such a large sample set. The main signs of CVL considered were onychogryphosis, ophthalmologic abnormalities, adenitis, cachexia, hepatosplenomegaly, desquamation, and crusted ulcers; dogs were classified as asymptomatic (the absence of clinical signs), oligosymptomatic (the presence of one to three clinical signs), or symptomatic (the presence of more than three clinical signs according to the criterion adapted from Mancianti et al.).^19^

The samples were collected with the aim of building the National Serological Panel of Canine Visceral Leishmaniasis in Brazil during the period of 2008 to 2009. For this collection, dogs were gagged, mechanically contained, and sedated using ketamine hydrochloride (10 mg/kg) with acepromazine maleate (0.2 mg/kg). Subsequently, blood samples were collected from the jugular vein for serological evaluation. Fragments of healthy skin and, when present, of skin lesions were collected for parasitological culture, immunohistochemistry, and histopathology. Trichotomy using disposable stainless-steel blades, antisepsis, and 2% lidocaine as a local anaesthesia was performed prior to biopsy and collect cutaneous fragments. Four fragments of healthy skin were collected from the scapular region of each animal using a 3 mm punch. Two of these skin fragments were stored in sterile saline solution with antifungals and antibiotics for the isolation of the parasite in culture medium, according to the protocol by Madeira et al.^20^ The other two fragments were stored in 10% buffered formalin for histopathology (HP) and immunohistochemistry (IHC) according to Menezes et al.^21^

After sample collection and clinical evaluation in the field, the samples were immediately sent to our collaborating laboratories for the proposed analyses to be done within similar timeframes while respecting the work dynamics of each laboratory.

The parasites that were isolated in culture were characterised by isoenzymes using five enzymatic systems based on protocols previously defined by Cupolillo et al.: 6PGDH, GPI, NH, G6PDH, and PGM.^22^ The characterisation was performed to determine the species of CVL in each case and to positively identify cases of *L. infantum*.

Serological immunoassays were performed using DPP^®^ CVL kits according to the manufacturer’s recommendations.

Collected samples were taken to reference laboratory A, where the parasitological examinations were processed. Aliquots of the serum samples were prepared, stored at -70ºC, and then sent to three different laboratories: national reference laboratory A, state reference laboratory B, and municipal reference laboratory C. The samples were processed without noting the results of the parasitological tests to allow a blind analysis to be performed. The results of the clinical assessments and serological and parasitological examinations were statistically analysed independently in an epidemiology reference laboratory.

The results obtained were entered into a Microsoft Excel-Office^®^ spreadsheet. Based on the cross-distribution of positive and negative results in a 2x2 contingency table, sensitivity, specificity, accuracy, positive and negative predictive values, and the respective 95% confidence intervals (95% CI) were calculated with reference to the parasitological culture techniques, HP, and IHC. The sensitivity and specificity of DPP^®^ CVL were also analysed separating the animals into three groups based on their symptoms: asymptomatic, oligosymptomatic, and symptomatic.

As a reference standard for validation of the serological tests, dogs with at least one positive result in any of the three parasitological diagnostic tests were considered “cases” of *Leishmania* infection, whereas dogs with negative results for the three tests were considered “non-cases”.

*Ethics* - Study procedures were approved by the Ethics Committee on Animal Use (CEUA-FIOCRUZ) under license no. L-038/08.

## RESULTS

**Fig. 2:**
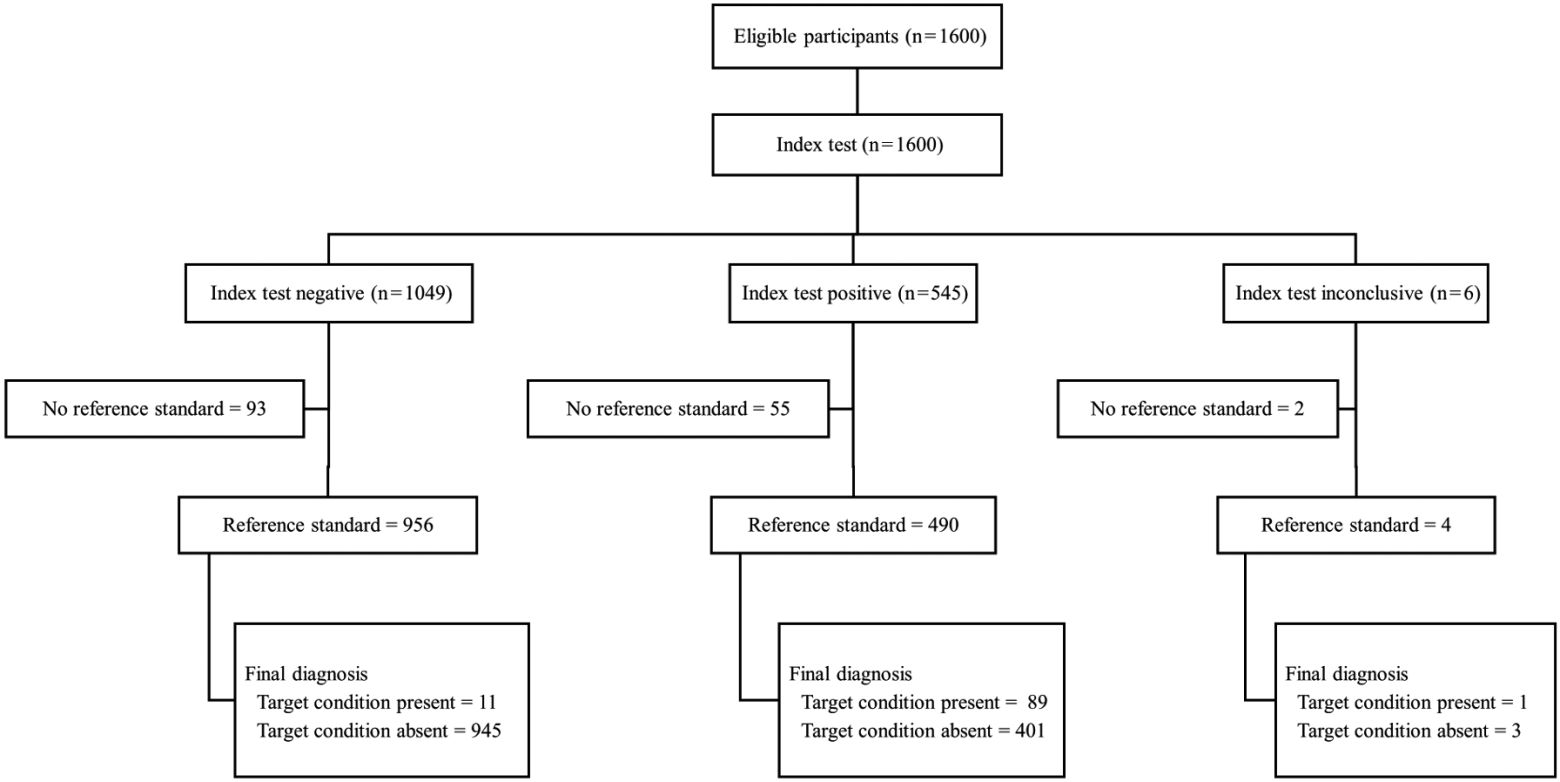
flow chart of the study participants with detailed information on the index and reference standard results

Fig. 2 shows the flow chart of the study participants with detailed information on the index and reference standard results. Table I presents in detail the infection prevalence, sensitivity, and specificity, as well as the positive and negative predictive values of the DPP^®^ immunoassays for all 1446 dogs in the sample set, both for the total canine population and the clinical subgroups (asymptomatic, oligosymptomatic, and symptomatic). The global prevalence of infection based on the reference standard was 6.9%, which increased with the presence of clinical signs of CVL. Positivity in the different parasitological tests was 4.0%, 3.8% and 5.5% for parasitological culture, histopathology, and immunohistochemistry, respectively. High overall sensitivity (89%) was observed. Sensitivity gradually decreased with the reduction of symptomatology in the animals and reached the lowest level (75%) in asymptomatic dogs. General specificity was 70%. Specificity gradually decreased with an increase in signs and symptoms in the dogs and reached the lowest level in symptomatic animals (56%).

As for inter-laboratory agreement, the Kappa (κ) indices obtained from the comparisons between the three participating laboratories (A, B, and C) were κ_AB_=0.778, κ_AC_=0.645 and κ_CB_=0.711; the concordance was substantial according to the classification by Landis and Koch.^23^

Table II shows the prevalence of infection, sensitivity, specificity, and positive and negative predictive values for the total canine population and clinical subgroups (asymptomatic, oligosymptomatic, and symptomatic) by municipality investigated. In general, consistent sensitivity and specificity results were observed between the municipalities, particularly with respect to increased sensitivity and decreased specificity as the analysed dogs presented more symptoms. The only anomaly was observed in Brasília, where sensitivity decreased as the symptoms increased. Nevertheless, the confidence intervals are quite broad, indicating a low precision in the estimates owing mainly to the small number of dogs with asymptomatic infection. In addition, as expected, the predictive values vary widely between municipalities because they depend directly on the prevalence values of canine infection.

**TABLE I:** Prevalence of infection, sensitivity, specificity, and positive/negative predictive values regarding the Dual-path Platform Chromatographic Immunoassay (DPP^®^ CVL) in a sample composed of 1446 dogs from areas endemic for canine visceral leishmaniasis assessed in single or clinical groups (asymptomatic, oligosymptomatic, and symptomatic)

Clinical condition	Number of dogs*	Prevalence of infection (95% CI) (%)	Sensitivity (95% CI) (%)	Specificity (95% CI) (%)	Positive predictive value (95% CI) (%)	Negative predictive value (95% CI) (%)
All (single group)	1446	6.9 (5.7-8.4)	89.0 (81.2-94.4)	70.2 (67.7-72.6)	18.2 (14.8-21.9)	98.8 (98.0-99.4)
Asymptomatic	448	2.7 (1.4-4.6)	75.0 (42.8-94.5)	72.9 (68.5-77.1)	7.1 (3.29-13.0)	99.1 (97.3-99.8)
Oligosymptomatic	721	5.1 (3.6-7.0)	89.2 (74.6-97.0)	70.3 (66.7-73.7)	14.0 (9.8-19.1)	99.2 (97.9-99.8)
Symptomatic	149	32.0 (25.0-40.4)	93.8 (82.8-98.7)	56.4 (46.2-66.3)	50.6 (39.8-61.3)	95.0 (86.1-99.0)

*: of the total number of dogs evaluated with valid information on parasitology and/or DPP (1446), only 1318 could be clinically assessed for classification into the asymptomatic, oligosymptomatic and symptomatic groups.

## DISCUSSION

The high overall sensitivity observed confirms the results of previous studies conducted with smaller sample sizes.^24^ ^,^ ^25^ In fact, the DPP^®^ CVL test was developed for joint detection of antibodies against K26 and K39 antigens,^26^ and historically, studies of the anti-*Leishmania* canine chromatographic immunoassay formulation have indicated an increased sensitivity when using both antigens together, while the use of k39 or rk39 in isolation has resulted in lower sensitivities.^27^ ^,^ ^28^ Subsequently, although Otranto et al.^29^ reached high sensitivity with the use of the rk39 antigen alone, other studies have suggested that the combined use of different antigens is associated with increased sensitivity in immunochromatographic tests;^17^ ^,^ ^25^ ^,^ ^30^ Souza Filho et al.^31^ demonstrated high sensitivity with the use of the Alere^TM^ test, which also utilises chimaera rK28. High sensitivity is a characteristic required when using diagnostic tests as a screening tool for the Visceral Leishmaniasis Control Programme.^32^

The parasitological methods used in this study are considered the gold standards for leishmaniasis diagnosis.^14^ Despite some limitations, such as the need for pathologist expertise in microscopic amastigote detection^33^ and its susceptibility to contamination, parasitological diagnosis is still considered the best method for diagnosis because of its high specificity.^14^ However, even considering that we used three different parasitological tests to build our reference standard, one should keep in mind that the results of the study might be slightly biased due to the well-known imperfections in the sensitivity of such tests.

The results of the clinical group evaluations showed a gradual decrease in test sensitivity accompanying the reduction of clinical signs in the dogs. Indeed, studies conducted using methodologies based on detection of serological response present high sensitivity and specificity in symptomatic dogs.^34^ However, it is worth noting that the mean sensitivity found in asymptomatic animals is still considerably higher than that observed in asymptomatic dogs in studies with smaller sample sizes, in which sensitivity was close to 50% as determined by the DPP^®^ CVL test or similar immunoassays using rk39 antigen;^17^ ^,^ ^35^ the assessment of this study also showed high negative predictive values. Such variation, in comparison with symptomatic dogs, is probably explained by the fact that the latter present high levels of non-protective antibodies,^36^ ^,^ ^37^ which would facilitate their detection, whereas the lower antibody levels detected in asymptomatic animals influence the accuracy of the serological methodologies.^34^ It should be noted that the sensitivity of chromatographic immunoassays may vary according to the course of infection.^38^

Thus, the large-scale assessment performed in this study demonstrates that symptomatology affects test performance but suggests that such influence occurs in a smaller proportion of tests than previously observed. Indeed, favourable results can also be found in asymptomatic animals. Recently, Larson et al.^39^ demonstrated that most animals, whether symptomatic or asymptomatic, tested positive in less than 3 min when the response time of the DPP^®^ CVL test was measured. Laurenti et al.^25^ detected infection of both symptomatic and asymptomatic animals in equal proportions. However, such findings do not eliminate the need for caution when assessing asymptomatic dogs, and the results of rapid tests, especially negative ones, should generally be evaluated with caution.^26^

Accordingly, the use in parallel (jointly) of enzyme-linked immunosorbent assay (ELISA) can increase the sensitivity of the assessment.^24^ This assay is already used serially as a confirmatory test for CVL according to the Brazilian Ministry of Health protocol.^15^ Regarding the application of this protocol, Coura-Vital et al.^40^ demonstrated an increase in CVL detection, in relation to prevalence and incidence measurements, when DPP^®^ CVL was utilised jointly with ELISAs as opposed to the previously used immunofluorescence technique. Nevertheless, there is discussion of reversing the protocol order, especially in locations with great diagnostic demand; such discussion suggests the use of ELISAs as a screening method and DPP^®^ CVL for confirmation because of the high specificity and positive predictive value previously reported for DPP^®^ CVL and aims to reduce the costs and increase the quality control of evaluation.^25^ ^,^ ^40^ However, the results of this survey showed a relative reduction in specificity, as well as in positive predictive value, when compared with studies conducted with smaller sample sets,^24^ ^,^ ^25^ which indicates a need for caution in the face of such propositions.

**TABLE II:** Prevalence of infection, sensitivity, specificity, and positive/negative predictive values regarding the Dual-path Platform Chromatographic Immunoassay (DPP^®^ CVL) in a sample of 1446 dogs from areas endemic for canine visceral leishmaniasis assessed in single or clinical groups (asymptomatic, oligosymptomatic, and symptomatic) according to the municipality investigated

Municipality	Clinical condition	Number of dogs*	Prevalence of infection (95% CI) (%)	Sensitivity (95% CI) (%)	Specificity (95% CI) (%)	Positive predictive value (95% CI) (%)	Negative predictive value (95% CI) (%)
Fortaleza	All (single group)	333	7.5 (4.9-10.9)	92.0 (74.0-99.0)	71.8 (66.4-76.7)	20.9 (13.7-29.7)	99.1 (96.8-99.9)
	Asymptomatic	134	3.7 (1.2-8.5)	60.0 (14.7-94.7)	72.9 (64.3-80.3)	7.9 (1.7-21.4)	97.9 (92.7-99.7)
	Oligosymptomatic	132	3.0 (0.8-7.6)	100.0 (39.8-100.0)	71.9 (63.2-79.5)	10.0 (2.8-23.7)	100.0 (96.1-100.0)
	Symptomatic	36	39.0 (23.0-56.5)	100.0 (76.8-100.0)	45.5 (24.4-67.8)	53.8 (33.4-73.4)	100.0 (69.2-100.0)
Palmas	All (single group)	377	2.7 (1.3-4.8)	100.0 (69.0-100.0)	59.4 (54.2-64.5)	6.3 (3.1-11.3)	100.0 (98.3-100.0)
	Asymptomatic	129	1.6 (0.2-5.5)	100.0 (15.8-100.0)	64.6 (55.6-72.8)	4.3 (0.5-14.5)	100.0 (95.6-100.0)
	Oligosymptomatic	179	2.2 (0.6-5.6)	100.0 (39.8-100.0)	57.1 (49.5-64.6)	5.1 (1.4-12.5)	100.0 (96.4-100.0)
	Symptomatic	16	25.0 (7.3-52.4)	100.0 (39.8-100.0)	33.3 (9.9-65.1)	33.3 (9.9-65.1)	100.0 (39.8-100.0)
Bauru	All (single group)	379	11.0 (8.3-15.0)	83.7 (69.3-93.2)	70.5 (65.3-75.4)	26.7 (19.4-35.0)	97.1 (94.2-98.8)
	Asymptomatic	76	3.9 (0.8-11.1)	66.7 (9.4-99.2)	69.9 (58.0-80.1)	8.3 (1.0-27.0)	98.1 (89.7-100.0)
	Oligosymptomatic	220	7.3 (4.2-11.5)	81.3 (54.4-96.0)	72.5 (65.9-78.5)	18.8 (10.4-30.1)	98.0 (94.3-99.6)
	Symptomatic	66	35.0 (24.0-47.6)	91.3 (72.0-98.9)	67.4 (51.5-80.9)	60.0 (42.1-76.1)	93.5 (78.6-99.2)
Brasília	All (single group)	357	6.2 (3.9-9.2)	90.9 (70.8-98.9)	80.3 (75.6-84.4)	23.3 (14.8-33.6)	99.3 (97.4-99.9)
	Asymptomatic	109	1.8 (0.2-6.5)	100.0 (15.8-100.0)	85.0 (76.9-91.2)	11.1 (1.4-34.7)	100.0 (96.0-100.0)
	Oligosymptomatic	190	6.8 (3.7-11.4)	92.3 (84.0-99.8)	79.7 (73.0-85.3)	25.0 (13.6-39.6)	99.3 (96.1-100.0)
	Symptomatic	31	23.0 (9.6-41.1)	85.7 (42.1-99.6)	58.3 (36.6-77.9)	37.5 (15.2-64.6)	93.3 (68.1-99.8)

*: of the total number of dogs evaluated with valid information on parasitological and/or DPP (1446), only 1318 could be clinically assessed for classification into the asymptomatic, oligosymptomatic and symptomatic groups.

It is also important to highlight that the DPP^®^ sequence as a screening method with ELISA as a confirmatory test is appropriate to the reality of small municipalities unable to maintain a laboratory for the performance of ELISAs, which can only be performed in central laboratories to confirm the diagnosis.^40^ In this context, the DPP^®^ CVL test is a screening tool that is easy to store, transport, and use and is able to achieve simple and fast results without the need of specialised laboratories.^24^ In addition, the substantial agreement between the three participating laboratories in a large-scale blind analysis demonstrates the reproducibility of the results and confirms the ease of use of the DPP^®^ CVL assay, which decreased execution errors.

Another aspect worth mentioning is the necessity to verify, prior to the diagnostic test, any possible anti-*Leishmania* vaccination of the dogs, considering that serological tests may not distinguish between infected and vaccinated animals.^41^ Studies have diverged with regard to the results obtained on cross-reactivity: Campos et al.^42^ recently demonstrated no cross-reactivity of DPP^®^ CVL for up to 12 months after vaccination of animals in a non-endemic area, whereas Marcondes et al.^43^ reported that the test can cross-react with vaccine antibodies for up to six months after vaccination. Therefore, such information must be considered before the interpretation of test results.

A comprehensive assessment of possible cross-reactivity, which the method is subject to, is also suggested.^34^ The results in the literature are still contradictory, presenting studies that did not observe cross-reactivity^24^ ^,^ ^44^ as well as surveys that demonstrated cross-reactivity with canine babesiosis^25^ and *Leishmania braziliensis*.^17^

Ultimately, Schubach et al.^32^ used data from one of the cities enrolled in our four-city study (namely, Fortaleza) to evaluate the performance of the rapid test and found comparable accuracy values using whole blood and serum samples through electronic or visual readings. Although they used some of the data from our study, it should be noted that our study does not focus on the stability of the results between types of samples. We used a much larger sample to evaluate accuracy and reliability of the test, as well as how this relates to the presence of CVL clinical signs. It is strongly recommended, however, that future systematic reviews in this field do not include both papers as if they used completely different sample sets.

In conclusion, DPP^®^ CVL performance is altered according to canine symptomatology, but such influence was less evident than in previous studies. Favourable results for sensitivity and specificity can be obtained even in asymptomatic animals; however, caution is needed in these evaluations, and the results suggest that immunochromatographic assays may be further improved for better investigation in asymptomatic dogs. However, the results obtained confirm the usefulness of DPP^®^ CVL for application in serological surveys.
